# Does Environmental Regulation Help Mitigate Factor Misallocation?—Theoretical Simulations Based on a Dynamic General Equilibrium Model and the Perspective of TFP

**DOI:** 10.3390/ijerph19063642

**Published:** 2022-03-18

**Authors:** Xu Dong, Yali Yang, Qinqin Zhuang, Weili Xie, Xiaomeng Zhao

**Affiliations:** 1 School of Economics, Zhengzhou University of Aeronautics, Zhengzhou 450046, China; dongx2018@zua.edu.cn (X.D.); xieweili0906@163.com (W.X.); 2 School of Information Management, Zhengzhou University of Aeronautics, Zhengzhou 450046, China; littleironer@163.com; 3 Institute of Quantitative & Technical Economics, Chinese Academy of Social Sciences, Beijing 100732, China; zhuangqinqin@cass.org.cn; 4 School of Economics and Business Administration, Central China Normal University, Wuhan 430079, China

**Keywords:** environmental regulation, factor misallocation, total factor productivity, dynamic general equilibrium model, numerical simulation

## Abstract

How environmental regulation affects factor allocation is becoming an emerging hot topic in academia. In this paper, we construct a dynamic general equilibrium model accommodating environmental regulatory shock based on the H-K framework to explain the impact of environmental regulation on factor misallocation from the perspective of aggregate total factor productivity loss changes, and numerical simulation results are provided for several representative scenarios. The results show that environmental regulation has a significant effect on factor market misallocation, but this effect is not simply positive or negative, and it mainly depends on the firms’ initial factor allocation status and the intensity of the shock. Reducing the intensity of environmental regulation for firms that face stronger distortion helps mitigate factor misallocation and, on the contrary, the same policy could exacerbate factor market misallocation. Under the environmental regulatory shock condition, firms’ overhead labor input has a moderating effect on the factor allocation mitigation of environmental regulation. Distorted firms’ higher overhead labor share inhibits the correction of factor misallocation by environmental regulation. And reducing firms’ overhead labor share amplifies the correcting effect of environmental regulation on factor misallocation.

## 1. Introduction

How to allocate factors of production is a core issue in economics and social science, and how to improve the efficiency of factor allocation is also an important topic concerned by academia and policymakers [1]. Factor markets usually suffer from widespread misallocation due to market failures, inadequate institutions, and imperfect policies, resulting in significant losses to macroeconomic development efficiency. Numerous theoretical and empirical studies have confirmed this phenomenon. For instance, Hsieh and Klenow (2009) found considerable factor misallocation in the manufacturing sector in China and India, with losses to TFP of 30–50% and 40–60%, respectively [2]. A study by Bartelsman et al. (2013) also found that factor misallocation exists in Europe and the United States, with the degree of misallocation in some Eastern European countries is much higher than that in Western Europe and the United States (about 3–7 times higher than in Western Europe and 10 times higher than in the United States) [3]. Given the above, the study of factor misallocation has become one of the key issues in the field of factor allocation. Especially, exploring the influencing factors of misallocation and their mechanism has been the focus of attention.

With the gradual increase of environmental costs in the process of economic development, governments have started to restrain market players through environmental regulation [4], and related academic research has been increasing. Early studies focused on the impact of environmental regulation on firms’ productivity with the established assumption that the impact of environmental regulation is symmetric for all firms, and then regulation can only have an impact by affecting factor re-organization and technological innovation within firms, thus arguing that environmental regulation does not have the function of allocating factors of production [5,6,7]. However, theories related to environmental economic geography pointed out that these studies ignored the issue of factor market distortions under the influence of environmental regulation that would significantly affect the productivity of industries and even macroeconomics, i.e., the idea that environmental regulation has a factor allocation effect [8,9,10,11]. So, what is the effect of environmental regulation on factor misallocation? How does the mechanism work? How to identify the magnitude of the impact? In this paper, we will answer the above questions through in-depth theoretical derivations and numerical simulations.

The rest of the paper proceeds as follows. We will review some relevant literature in Section 2 and construct a theoretical model in Section 3. Based on the model, Section 4 illustrates the impact of environmental regulation on factor misallocation by numerical simulations. Finally, Section 5 provides the conclusions and implications.

## 2. Literature Review

Studies on the impact of environmental regulation on factor misallocation belong to the intersection of environmental policy and factor allocation. The research directions that are highly relevant and supportive to this paper broadly include the following two: the source of factor misallocation and the effects of environmental regulation.

### 2.1. The Sources of Factor Misallocation

As regards the sources of factor misallocation, the prevailing view is that various types of distortions are the main causes. By analyzing the existing literature, we summarize the sources of factor misallocation as market-based, institutional, integrative, and other factors.

Market-based factors are mainly reflected in the incompleteness of factor markets. Due to the frequent occurrence of financial frictions, credit discrimination, and banking system dysfunctions, the financial market is not perfect but suffers from widespread distortions that lead to capital misallocation [12,13,14]. Similar to the financial market, widespread information asymmetry, monopoly power, and gender discrimination in the labor market are common causes of labor misallocation [15,16,17]. In addition, the sources of land, energy, and technology misallocation have also been discussed around incomplete land, energy, and technology markets [18,19], and their specific causes and transmission mechanisms are similar to the formation of capital and labor misallocation.

Industrial policy distortion and unsound mechanism design are the main institutional factors. It has been shown that biased industrial policies implemented by the government through subsidies, taxation, debt extension, and scale control not only cause a wasteful and inefficient allocation of resources but also seriously interfere with the nor-mal market meritocracy [20,21]. Some scholars have examined the impact of the household registration system on factor allocation from the perspectives of employment choice and investment decisions, arguing that the household registration system severely constrains the efficient allocation of labor resources in the market [22]. Also, some other scholars pointed out that an un-sound property rights protection system was an important cause of factor misallocation based on the relationship between property rights protection and economic development [23].

In some studies, the role of market-based and institutional factors on resource misallocation is not independent but intertwined, especially in the fields of international trade and infrastructure. The resource misallocation caused by international trade includes both market-based factors such as “iceberg cost”, and institutional factors such as tariffs, quotas, and other trade barriers [24,25,26]. In comparison to studies of resource misallocation in the international trade pattern, some scholars have turned their perspectives to the domestic market and found that the differences in infrastructure conditions in different regions can also lead to resource misallocation under the dual role of market and institution [27].

In addition to the more discussed distortions mentioned above, some literature also discussed the effects of factors such as firm establishment, zombie firms, and high housing prices on factor mismatch. Hsieh and Klenow (2009) and Shao et al. (2013) argued that different years of a firm establishment could lead to factor misallocation within industries [2,28]. Zhang et al. (2019) found that the larger the asset share and number share of zombie firms, the larger the loss caused by factor misallocation at the urban level [29]. Chen et al. (2015) investigated the factor misallocation caused by high housing prices and concluded that the “in-verse” mechanism of corporate profits and total factor productivity due to high housing prices is an important cause of factor misallocation [30].

### 2.2. The Effects of Environmental Regulation

Currently, academia generally believes that environmental regulation has multidimensional effects and is an important policy tool to achieve sustainable development [31,32,33]. Summarily, there is a growing diversity of research topics on the effects of environmental regulation, covering environmental, economic, and social effects.

In terms of causality, environmental regulation arises in response to the growing problem of environmental pollution, and thus the environmental effect is an inherent policy effect of environmental regulation [34]. On the one hand, effective environmental regulation can force polluting firms to reduce their pollution or bring them into compliance by requiring firms to adopt green technology, thus improving environmental quality to some extent [35,36]. On the other hand, strict environmental regulation usually leads to an increase in production costs for firms, which may choose to move to areas with less stringent environmental regulation, the so-called “pollution haven”. The process is accompanied by a large-scale transfer of pollutants, thus causing damage to the environment in the places the firms move to [37,38,39,40]. In addition, some other studies have shown that the environmental effects of environmental regulation are nonlinear [41].

In the economic development sphere, the impact of environmental regulation can be gradually transmitted from firms to industries and the wider economy. At the micro-level, there are two mainstream theoretical hypotheses on the effects of environmental regulation—the “Porter hypothesis” and “race to the bottom hypothesis”. The Porter hypothesis believes that in the short run, environmental regulation causes an increase in production costs and harms the productivity of firms and industries [42,43,44], but in the long run, it can motivate firms to strengthen technological innovation and generate innovation compensation effect, which can partially or even fully offset the cost increase caused by environmental regulation, to improve the productivity of enterprises, industries and even macroeconomics [5,7,45]. The race to the bottom hypothesis mainly portrays the impact of environmental regulation on firms’ location decisions and business strategies, etc., and argues that the probability of firms’ proximity location shift is greatly increased under the increasingly stringent environmental regulation. At the same time, firms’ business decision-making also changes to adapt to changes in environmental regulation policies [46,47,48]. At the macro level, the economic effects of environmental regulation are mainly reflected in the effects on economic growth [49], industrial structure upgrading [50,51], total factor productivity [52,53], foreign trade, and outward investment attraction [54,55,56], etc. In terms of research findings, most scholars’ views hold that the macroeconomic effects of environmental regulation are characterized by great uncertainty or nonlinearity, i.e., the direction of the impact of environmental regulation on macroeconomic development may be positive or negative; the trend of the impact may be U-shaped or inverted U-shaped and/or other more complex trends.

Relatively few studies have been conducted on the social effects of environmental regulation, which are based on the expansion and deepening of environmental and economic effects. It has been pointed out that environmental regulation can effectively reduce the health risks of the population by improving environmental quality [57]. At the same time, environmental regulation may also have different influences on labor employment through “crowding-out effects” and “substitution effects” [58]. On the one hand, environmental regulation may typically lead to higher production costs for firms, forcing them to curtail labor demand or use more affordable labor [59,60,61]. On the other hand, environmental regulations may also motivate firms to engage in green innovation, thus creating and providing more green jobs [62,63]. Furthermore, environmental regulations may have a shock on the income level and mobility of the population by affecting labor employment [64].

### 2.3. The Brief Comments

In summary, the growing research literature in the fields of the sources of factor misallocation and the effects of environmental regulation has paved the way for our work. However, it is also clear that there is little literature that explores the causes of factor misallocation from the perspective of environmental regulation. Similarly, although there is literature confirming that environmental regulation has factor allocation effects, it is relatively rare to directly explore whether and how environmental regulation affects factor misallocation, which is the focus of our paper.

The limited literature that directly investigated the impact of environmental regulations on factor misallocation focused on empirical studies, but the conclusions reached were inconsistent due to differences in sample data, estimation strategies, and methods, and two opposite views have emerged. One view is that environmental regulation increases the cost of production and distorts factor allocation by causing some factors of production to flow to sectors or regions with more lenient regulatory policies [10,65,66]. The other view points out that environmental regulation may lead to higher production costs in the short run, but the resulting “innovation compensation” will be beneficial to factor allocation efficiency in the long run [5,67,68]. In addition, some scholars have also pointed out that studying the factor allocation effects of environmental regulation needs to consider the heterogeneity of industries and regions [69,70]. To date, studies that examine the impact of environmental regulation on factor misallocation from the theoretical dimensions have not been found.

To fill the research shortage in this field, we attempt to construct a dynamic general equilibrium model that accommodates environmental regulatory shock, to theoretically explain how environmental regulation affects factor misallocation, and to measure and visualize the effect of environmental regulation on factor misallocation through numerical simulation. Our work not only helps to enrich the research related to the impact effects of environmental regulation but also is an important extension of the study on the sources of factor misallocation, which constitutes an important difference and innovation of this paper relative to the existing literature.

## 3. The Model

**Figure 1:**
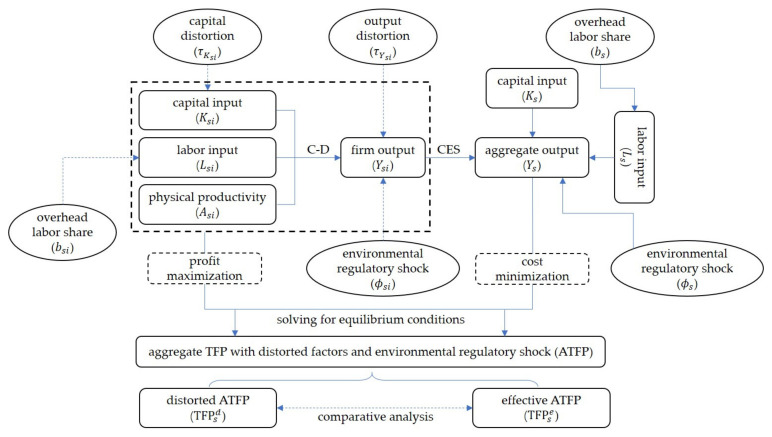
Schematic diagram of the logical relationships of the model.

In this section, we try to construct a dynamic general equilibrium model with environmental policy interventions, where environmental regulation is introduced as an external shock in a “micro (firm) to macro (aggregate)” analytical framework. The model is constructed by starting from the product market equilibrium and then introducing the market distortion and environmental regulation shock to solve the equilibrium solution under the optimality condition to obtain distorted and effective aggregate total factor productivity. On this basis, we determine whether environmental regulation helps to mitigate factor misallocation by comparing whether the loss of factor market distortions to aggregate TFP decreases in the presence and absence of environmental regulatory shock. Following Hsieh and Klenow (2009), we take a relative level approach to define factor market distortions by denoting output distortions by $\tau_{Y}$ and capital distortions by $\tau_{K}$, respectively [2] (Output distortion is described in relative level as equivalent to labor distortion in absolute level, see Hsieh and Klenow (2009) for details. In this sense, output distortion and capital distortion defined in this paper actually reflect the misallocation of the labor and capital markets, respectively). The former reflects distortions caused by the same proportional increase in marginal output of capital and labor factors, and the latter represents distortions caused by the increase in marginal output of capital relative to labor factors. Unusually, we will also include overhead labor as adjustment friction to observe its impact on the robustness of the model. The difference is that we will also include overhead labor as adjustment friction to observe its impact on the model. Figure 1 shows the main components of the model and the logical relationships between the variables involved in it.

### 3.1. Product Market Equilibrium

Assume that the final product market in any region is perfectly competitive and has only one product Y, which is obtained by weighting the output $Y_{s}$ of S industries, i.e.,

(1) $$Y=\underset{s=1}{\prod^{S}}Y_{s}^{\theta_{s}}$$

where $\theta_{s}$ denotes the output of the sth industry as a share of the total output of the economy. Under perfect competition, the cost minimization principle yields

(2) $$P_{s}Y_{s}=\theta_{s}PY$$

where $P_{s}$ is the price of output of the sth industry and P is the price of the final product. Since the final product is generally set as a valued good, P=1. Any industry s is assumed to consist of I monopolistically competitive firms whose output $Y_{s}$ is a constant elasticity of substitution (CES) function summed over the output $Y_{si}$ of these firms, i.e.,

(3) $$Y_{s}={\left(\underset{i=1}{\sum^{I}}Y_{si}^{\frac{\sigma -1}{\sigma }}\right)}^{\frac{\sigma }{\sigma -1}}$$

where σ>1 is the product elasticity of substitution among firms in the industry.

The production function of a representative monopolistically competitive firm as an intermediate good producer in industry s subject to environmental regulatory shock is assumed to be given by the Cobb-Douglas form, i.e.,

(4) $$Y_{si}=\phi_{si}A_{si}K_{si}^{\alpha }{\left[\left(1-b_{si}\right)L_{si}\right]}^{1-\alpha }$$

where $A_{si}$, $K_{si}$ and $L_{si}$ denote the technology level, capital input, and labor input of the ith firm in industry s in the production process, respectively; $b_{si}$ denotes the share of overhead labor in a firm’s total labor input, which can reflect the extent to which the firm’s production is affected by adjustment frictions; $\phi_{si}$ is the environmental regulatory shock to the firm, which is usually quantified by the intensity of regulation, so $\phi_{si}>0$; α is the output elasticity of capital, and in this paper, we assume that the return of scale is constant and the corresponding labor output elasticity is (1−α). In addition, we also assume that the output elasticities of capital and labor are the same among firms within the same industry.

In the presence of distortions in factor markets, different firms are subject to different degrees of distortionary shocks due to differences in their factor endowments and operating capabilities, which cause the factor marginal output to deviate from the optimal growth path. As defined by Hsieh and Klenow (2009), output distortions increase the marginal output of capital and labor in the same proportion, which is effectively equivalent to taxing firms’ output $Y_{si}$; capital distortions raise the marginal output of capital relative to labor, which is equivalent to taxing firms’ investment behavior [2]. But in any case, the firm still aims at profit maximization, and its optimization problem can be expressed as follows.

(5) $$\begin{array}{c} \max\pi_{si}\;=\;\left(1-\tau_{Y_{si}}\right)P_{si}Y_{si}\;-\;\left(1+\tau_{K_{si}}\right)rK_{si}\;-\;\left(1-b_{si}\right)wL_{si}\\ s.t.\;Y_{si}={\left(\frac{P_{s}}{P_{si}}\right)}^{\sigma }Y_{s} \end{array}$$

where r and w denote the unit cost of capital (interest rate) and labor (real wage) to be paid by the firm in the production process, respectively. To simplify the analysis, we assume that both the unit cost of capital and unit cost of labor are the same across firms, which is consistent with Hsieh and Klenow (2009) [2].

Solving the first-order condition for the firm’s profit maximization problem represented by Equation (5), we can obtain the equilibrium price of the firm’s output as a fixed price additive to its unit factor input cost, i.e.,

(6) $$P_{si}=\frac{\sigma }{\sigma -1}\frac{1}{1-\tau_{Y_{si}}}{\left(\frac{r}{\alpha }\right)}^{\alpha }{\left(\frac{w}{1-\alpha }\right)}^{1-\alpha }\frac{{\left(1+\tau_{K_{si}}\right)}^{\alpha }}{\phi_{si}A_{si}}$$

Meanwhile, the level of output and factor allocation of firms in equilibrium can be obtained according to the first-order conditions.

(7) $$Y_{si}\propto \frac{{\left(\phi_{si}A_{si}\right)}^{\sigma }{\left(1-\tau_{Y_{si}}\right)}^{\sigma }}{{\left(1+\tau_{K_{si}}\right)}^{\alpha \sigma }}$$

(8) $$K_{si}\propto \frac{{\left(\phi_{si}A_{si}\right)}^{\sigma -1}{\left(1-\tau_{Y_{si}}\right)}^{\sigma }}{{\left(1+\tau_{K_{si}}\right)}^{\alpha \left(\sigma -1\right)+1}}$$

(9) $$L_{si}\propto \frac{{\left(\phi_{si}A_{si}\right)}^{\sigma -1}{\left(1-\tau_{Y_{si}}\right)}^{\sigma }}{\left(1-b_{si}\right){\left(1+\tau_{K_{si}}\right)}^{\alpha \left(\sigma -1\right)}}$$

Equations (6) and (7) show that factor market distortions cause the level of output and prices of firms to deviate from the level that it would be if factor allocation were efficient. For a given wage and interest rate, output distortion and capital distortion exacerbates the decline in the level of output and the increase in the price of output, but the effects caused by distortions are likely to be somewhat intervened or moderated by the presence of environmental regulatory shock.

On the other hand, Equations (8) and (9) show that the factor allocation of the firm in equilibrium is affected by both output distortions and capital distortions, which will lead to differences in the marginal output of capital and labor across firms. The misallocation makes the marginal output of capital proportional to the firm’s unit cost of capital and the marginal output of labor proportional to the firm’s unit cost of labor, i.e.,

(10) $${\mathrm{MRPK}}_{si}=r\frac{1+\tau_{K_{si}}}{1-\tau_{Y_{si}}}$$

(11) $${\mathrm{MRPL}}_{si}=w\frac{1-b_{si}}{1-\tau_{Y_{si}}}$$

### 3.2. Factor Misallocation and TFP Losses Shocked by Environmental Regulation

Factor allocation is efficient if the marginal revenue output of a firm’s capital and labor are equal. Otherwise, it is considered factor misallocation. Factor misallocation caused by market distortions can have a direct impact on the size of a firm’s revenue, which in turn affects the firm’s TFP. The impact of factor market distortions on firm revenue size is discussed using a two-firm model as an example. According to the intermediate product market equilibrium, a firm’s output is a Cobb-Douglas function of its production technology, output distortions, capital distortions, and environmental regulatory shock. To simplify the analysis, it is assumed that capital distortions remain constant, and firm 1 faces output distortion while firm 2 does not have output distortions. Based on this, from Equation (7), it follows

(12) $$\frac{Y_{s1}}{Y_{s2}}={\left(\frac{\phi_{s1}A_{s1}}{\phi_{s2}A_{s2}}\right)}^{\sigma }{\left(1-\tau_{Y_{s1}}\right)}^{\sigma }<\frac{Y_{s1}^{e}}{Y_{s2}}$$

where $Y_{s1}^{e}$ denotes the output of firm 1 when the factor allocation is efficient. Equation (12) shows that the presence of output distortion will cause the output of firm 1, which faces factor allocation distortion, to be lower than its efficient level. Meanwhile, according to Equation (6), the output price ratio of firm 1 to firm 2 is obtained as

(13) $$\frac{P_{s1}}{P_{s2}}=\frac{\phi_{s2}A_{s2}}{\phi_{s1}A_{s1}\left(1-\tau_{Y_{s1}}\right)}$$

Furthermore, the revenue size ratios of firm 1 and firm 2 are obtained from Equations (12) and (13), i.e.,

(14) $$\frac{P_{s1}Y_{s1}}{P_{s2}Y_{s2}}={\left(\frac{\phi_{s1}A_{s1}}{\phi_{s2}A_{s2}}\right)}^{\sigma -1}{\left(1-\tau_{Y_{s1}}\right)}^{\sigma -1}<{\left(\frac{\phi_{s1}A_{s1}}{\phi_{s2}A_{s2}}\right)}^{\sigma -1}\frac{P_{s1}^{e}Y_{s1}^{e}}{P_{s2}Y_{s2}}$$

Equation (14) implies that the ratio of revenue size of firms with factor mismatch to those with efficient factor allocation and output distortion moves in the opposite direction. As the degree of output distortion rises, the difference in revenue size between firms increases.

According to Foster et al. (2008) and Hsieh and Klenow (2009), firm-level total factor productivity calculations can be distinguished into two types [2,71]: one is based on firm output and deflates output using a firm-level price index, called physical total factor productivity, denoted by TFPQ; the other is based on firm output revenue and deflates output based on macro price indices such as industry or region, called revenue total factor productivity, denoted by TFPR. By definition.

(15) $${\mathrm{TFPQ}}_{si}≜A_{si}=\frac{Y_{si}}{\phi_{si}K_{si}^{\alpha }{\left[\left(1-b_{si}\right)L_{si}\right]}^{1-\alpha }}$$

(16) $${\mathrm{TFPR}}_{si}≜P_{si}A_{si}=\frac{P_{si}Y_{si}}{\phi_{si}K_{si}^{\alpha }{\left[\left(1-b_{si}\right)L_{si}\right]}^{1-\alpha }}$$

In the absence of factor misallocation, more capital and labor should be allocated by market mechanisms to firms with higher TFPQ to the point where their higher output will lead to lower prices and have the same TFPR as smaller firms. A firm with higher TFPR implies that it faces barriers to raising the marginal products of labor and capital, resulting in the firm being smaller than optimal. With the help of Equations (10) and (11), the firm-level TFPR is obtained as the geometric average of its marginal output of capital and labor, i.e.,

(17) $${\mathrm{TFPR}}_{si}\propto {\mathrm{MRPK}}_{si}^{\alpha }{\mathrm{MRPL}}_{si}^{1-\alpha }$$

And then, the firm-level TFPR under the condition of factor misallocation is expressed in terms of some known parameters as

(18) $${\mathrm{TFPR}}_{si}\propto \frac{1+\tau_{K_{si}}}{\phi_{si}\left(1-\tau_{Y_{si}}\right)}$$

Equation (18) shows that TFPR is not intrinsically related to TFPQ, although they are very similar in form. Under the condition that the product substitution elasticity σ, capital-output elasticity α, the unit capital cost r and the unit labor cost w are given for firms in the industry, TFPR reflects the factor misallocation situation faced by firms. As the degree of capital distortion increases, firms can achieve higher TFPR; as output distortion increases, firms’ TFPR tends to decline. When there is no factor misallocation ($\tau_{K_{si}}=\tau_{YK_{si}}=0$), the TFPR of all firms is maintained at a fixed constant level, which is equivalent to the equilibrium state of the equal marginal output of factors among firms under the condition of no misallocation. On the other hand, it can also be seen from Equation (18) that environmental regulatory shock has an important impact on TFPR, which will keep decreasing as the degree of shocks increases. That is, to some extent, environmental regulatory shock can attenuate the negative impact of factor misallocation on firm productivity.

After obtaining the measure of firm-level TFP under the condition of factor misallocation, we proceed to derive industrial aggregate TFP (called ATFP below for ease of presentation) and express it as a function of the factor market distortion operator. On this basis, the impact of the environmental regulatory shock on factor misallocation.

According to the basic definition of total factor productivity, the aggregate total factor productivity ${\mathrm{TFP}}_{s}$ of industry s is equal to the residual value of industry output $Y_{s}$ after excluding industry capital input $K_{s}$ and labor input $L_{s}$. Analogously to the firm production function, considering the industry-level overhead labor share $b_{s}$ and environmental regulatory shock $\phi_{s}$, ATFP is expressed as

(19) $${\mathrm{TFP}}_{s}=\frac{Y_{s}}{\phi_{s}K_{s}^{\alpha }{\left[\left(1-b_{s}\right)L_{s}\right]}^{1-\alpha }}$$

From Equation (19), to express ATFP as a function of the factor market distortion operator, we must first obtain the total capital input $K_{s}$ and labor input $L_{s}$ under the condition of factor misallocation. Based on Equations (8) and (9), they can be easily written as follows

(20) $$K_{s}\propto \underset{i=1}{\sum^{I}}\frac{{\left(\phi_{si}A_{si}\right)}^{\sigma -1}{\left(1-\tau_{Y_{si}}\right)}^{\sigma }}{{\left(1+\tau_{K_{si}}\right)}^{\alpha \left(\sigma -1\right)+1}}$$

(21) $$L_{s}\propto \underset{i=1}{\sum^{I}}\frac{{\left(\phi_{si}A_{si}\right)}^{\sigma -1}{\left(1-\tau_{Y_{si}}\right)}^{\sigma }}{\left(1-b_{si}\right){\left(1+\tau_{K_{si}}\right)}^{\alpha \left(\sigma -1\right)}}$$

Substituting Equations (20) and (21) into Equation (19) and combining them with firm-level ${\mathrm{TFPR}}_{si}$ and solving for $P_{si}Y_{si}/P_{s}Y_{s}$, we obtain ATFP induced by factor misallocation under the environmental regulatory shock condition as

(22) $${\mathrm{TFP}}_{s}=\frac{\frac{1}{\phi_{s}{\left(1-b_{s}\right)}^{1-\alpha }}{\left[\sum_{i=1}^{I}{\left(\frac{\phi_{si}A_{si}\left(1-\tau_{Y_{si}}\right)}{{\left(1+\tau_{K_{si}}\right)}^{\alpha }}\right)}^{\sigma -1}\right]}^{\frac{\sigma }{\sigma -1}}}{{\left[\sum_{i=1}^{I}\frac{1-\tau_{Y_{si}}}{1+\tau_{K_{si}}}{\left(\frac{\phi_{si}A_{si}\left(1-\tau_{Y_{si}}\right)}{{\left(1+\tau_{K_{si}}\right)}^{\alpha }}\right)}^{\sigma -1}\right]}^{\alpha }{\left[\sum_{i=1}^{I}\frac{1-\tau_{Y_{si}}}{1-b_{si}}{\left(\frac{\phi_{si}A_{si}\left(1-\tau_{Y_{si}}\right)}{{\left(1+\tau_{K_{si}}\right)}^{\alpha }}\right)}^{\sigma -1}\right]}^{1-\alpha }}$$

## 4. Numerical Simulation

Based on the theoretical model mentioned above, we will discuss the effect of environmental regulation on factor misallocation through numerical simulations in this section. The basic idea is to observe how TFP loss due to factor misallocation changes in response to an environmental regulatory shock. If the industrial ATFP loss narrows due to factor misallocation at the firm level under the effect of environmental regulatory shock, then environmental regulation helps to suppress factor misallocation. Conversely, environmental regulation may lead to an exacerbation of factor misallocation. To simplify the analysis, we assume that there are only two competitive firms in the industry, where firm 1 faces factor misallocation while firm 2 has efficient factor allocation. Based on Equation (22), ${\mathrm{TFP}}_{s}^{d}$ is used to denote the distorted aggregate total factor productivity and ${\mathrm{TFP}}_{s}^{e}$ represents effective total factor productivity for the two-firm case. The expressions are shown as the following (For Equation (23), we assumed the existence of factor misallocation for firm 1 but not for firm 2. Therefore, for the sake of simplicity in the form of the equation, the output distortion and capital distortion for firm 1 are abbreviated as $\tau_{Y}$ and $\tau_{K}$ respectively):

(23) $${\mathrm{TFP}}_{s}^{d}\;=\;\frac{\frac{1}{\phi_{s}{\left(1\;-\;b_{s}\right)}^{1-\alpha }}{\left[{\left(\frac{\phi_{s1}A_{s1}\left(1\;-\;\tau_{Y}\right)}{{\left(1\;+\;\tau_{K}\right)}^{\alpha }}\right)}^{\sigma -1}+{\left(\phi_{s2}A_{s2}\right)}^{\sigma -1}\right]}^{\frac{\sigma }{\sigma -1}}}{{\left[\frac{1\;-\;\tau_{Y}}{1\;+\;\tau_{K}}{\left(\frac{\phi_{s1}A_{s1}\left(1\;-\;\tau_{Y}\right)}{{\left(1\;+\;\tau_{K}\right)}^{\alpha }}\right)}^{\sigma -1}+{\left(\phi_{s2}A_{s2}\right)}^{\sigma -1}\right]}^{\alpha }{\left[\frac{1\;-\;\tau_{Y_{si}}}{1\;-\;b_{s1}}{\left(\frac{\phi_{s1}A_{s1}\left(1\;-\;\tau_{Y}\right)}{{\left(1\;+\;\tau_{K}\right)}^{\alpha }}\right)}^{\sigma -1}\;+\;\frac{{\left(\phi_{s2}A_{s2}\right)}^{\sigma -1}}{1\;-\;b_{s2}}\right]}^{1-\alpha }}$$

(24) $${\mathrm{TFP}}_{s}^{e}\;=\;\frac{\frac{1}{\phi_{s}{\left(1\;-\;b_{s}\right)}^{1-\alpha }}{\left[{\left(\phi_{s1}A_{s1}\right)}^{\sigma -1}\;+\;{\left(\phi_{s2}A_{s2}\right)}^{\sigma -1}\right]}^{\frac{\sigma }{\sigma -1}}}{{\left[{\left(\phi_{s1}A_{s1}\right)}^{\sigma -1}\;+\;{\left(\phi_{s2}A_{s2}\right)}^{\sigma -1}\right]}^{\alpha }{\left[\frac{{\left(\phi_{s1}A_{s1}\right)}^{\sigma -1}}{1\;-\;b_{s1}}\;+\;\frac{{\left(\phi_{s2}A_{s2}\right)}^{\sigma -1}}{1\;-\;b_{s2}}\right]}^{1-\alpha }}$$

When a firm’s production is not affected by environmental regulatory shock and there is no overhead labor, environmental regulatory shock and overhead labor at the industry level will also not exist. In this case, Equations (23) and (24) are rewritten as follows, respectively.

(25) $${\mathrm{TFP}}_{s}^{d}\;=\;\frac{{\left[\frac{A_{s1}^{\sigma -1}{\left(1\;-\;\tau_{Y}\right)}^{\sigma -1}}{{\left(1\;+\;\tau_{K}\right)}^{\alpha \left(\sigma -1\right)}}\;+\;A_{s2}^{\sigma -1}\right]}^{\frac{\sigma }{\sigma -1}}}{{\left[\frac{A_{s1}^{\sigma -1}{\left(1\;-\;\tau_{Y}\right)}^{\sigma }}{{\left(1\;+\;\tau_{K}\right)}^{\alpha \left(\sigma -1\right)+1}}\;+\;A_{s2}^{\sigma -1}\right]}^{\alpha }{\left[\frac{A_{s1}^{\sigma -1}{\left(1\;-\;\tau_{Y}\right)}^{\sigma }}{{\left(1\;+\;\tau_{K}\right)}^{\alpha \left(\sigma -1\right)}}\;+\;A_{s2}^{\sigma -1}\right]}^{1-\alpha }}$$

(26) $${\mathrm{TFP}}_{s}^{e}\;=\;{\left(A_{s1}^{\sigma -1}\;+\;A_{s2}^{\sigma -1}\right)}^{\frac{1}{\sigma -1}}$$

### 4.1. Basic Assumptions and Parameter Settings

As shown in Equations (23) and (24), industrial ATFP in the two-firm case is affected by environmental regulatory shock and overhead labor share in addition to capital distortion and output distortion. Next, we will numerically simulate ATFP under the condition of factor misallocation with the presence and absence of environmental regulatory shock separately, and take overhead labor as a frictional factor into consideration. Before doing so, we need to assign values to some key parameters in the model. Following Hsieh and Klenow (2009) and Bartelsman et al. (2013) [2,3], we proceed as follows:

1. The elasticity of substitution between firm value-added is set to σ=3. Estimates of the substitutability among competing firms typically range from 3–10 in the vast literature on productivity studies [72,73]. Broda and Weinstein (2006) argued that lower elasticities for more differentiated goods, so we made this choice for σ conservatively [72]. Besides, we will also use a relatively moderate elasticity and a more extreme elasticity in simulations as robustness analysis. Table A1, Table A2, Table A3 and Table A4 in Appendix A show these results.
2. We set the elasticity of output with respect to capital for each firm to α=1/3, which is close to what has been found in most empirical studies [74,75].
3. As mentioned, we supposed that firm 1 in our model faced misallocation but not for firm 2. It is well known that misallocation leads to a loss of firm productivity, and thus we have good reasons to assume that the physical productivity of firm 1 is lower than firm 2. Based on this, we standardized the physical total factor productivity of the two firms as $A_{s1}=0.5$ and $A_{s2}=1$.
4. Environmental regulation is an external policy shock, thus, government tends to differentiate policies according to the wide heterogeneity of firms so that the extent of the shock may be different for different firms [76]. Firstly, we set a benchmark for the environmental regulatory shock—firm 1 and firm 2 face a unit shock at the same time, i.e., $\phi_{s1}=\phi_{s2}=1$. Secondly, we distinguish two alternative options: (1) firm 1 is subjected to a weaker environmental regulatory shock, defined as half the intensity of the benchmark; (2) firm 1 is subjected to a stronger environmental regulatory shock, defined as two times the intensity of the benchmark. Finally, assume that the industry-level environmental regulatory shock ($\phi_{s}$) is the geometric mean at the firm level.
5. Similar to the parameterization of environmental regulatory shock, we first consider a base case where firm1 and firm 2 have the same overhead labor share, set as $b_{s1}=b_{s2}=0.1$. Next, let the overhead labor share of firm 1 be half of the base case as one simulation option, and let the overhead labor share of firm 2 be half of the base case as another simulation option. In addition, the industry-level overhead labor share is also assumed as the firm-level geometric mean.

### 4.2. The Impacts of $\tau_{Y}$ on ATFP in the Absence/Presence of Environmental Regulatory Shock and Overhead Labor

According to Equations from (23) to (26), we will simulate the effects of environmental regulation on factor misallocation from the perspective of ATFP loss changes in the following six scenarios. Table 1 reports the effects of output distortion on the ATFP in six different scenarios. It can be seen that the presence of $\tau_{Y}$ always makes the distorted ATFP lower than the effective ATFP regardless of the scenarios, but the ATFP loss due to distortion is different when the environmental regulatory shock is present and absent, whereby we can judge the impact of environmental regulation on factor misallocation.

If environmental regulatory shock and overhead labor are not considered (Scenario 1), or when the setting for both is kept as the benchmark (Scenario 2), the effective ATFP was always 1.118, while the distorted ATFP decreased as output distortion raised. For example, given $\tau_{Y}=0.5$, the distorted ATFP was 1.062 and the corresponding ATFP loss was about 5.275%. The results of Scenario 2 also imply that the same proportion of environmental regulatory shock or overhead labor share to firms does not change the trend of ATFP.

When firm 1 is subject to a larger environmental regulatory shock than firm 2 (Scenario 3), both effective ATFP and distorted ATFP were lower than they would have been without the environmental regulatory shock or with the same degree of shock, and the output distortion leads to a larger decline in ATFP and a larger gap with effective ATFP. For example, given $\tau_{Y}=0.5$, the effective ATFP was 1.000, while the distorted ATFP was 0.878 and the corresponding ATFP loss was about 13.842%.

When firm 1 was subject to a smaller environmental regulatory shock than firm 2 (Scenario 4), both effective ATFP and distorted ATFP would be higher than they would have been in the absence of the environmental regulatory shock or with the same degree of shock, and the rate of decline in ATFP due to output distortion was slower. For example, given $\tau_{Y}=0.5$, the effective ATFP was 1.458, while the distorted ATFP was 1.436 and the corresponding ATFP loss was about 1.495%.

With the environmental regulatory shock to firms keeping the baseline case, if firm 1 has a higher share of overhead labor compared to firm 2 (Scenario 5), both effective ATFP and distorted ATFP are higher than they would be if firms’ overhead labor did not exist or had the same share, while distorted ATFP decreases at a slower rate with output distortion. And the difference between the distorted and effective ATFP tends to narrow. For example, given $\tau_{Y}=0.5$, the effective ATFP is 1.126 while the distorted ATFP is 1.077 and the corresponding ATFP loss is about 4.620%.

Likewise, in the baseline case of environmental regulatory shock holds, if firm 1 has a lower share of overhead labor compared to firm 2 (Scenario 6), the effective ATFP and distorted ATFP will both be lower than they would be if firms’ overhead labor was absent or the share was the same, while distorted ATFP will tend to decline more rapidly with the degree of output distortion. The gap between the distorted and effective ATFP will become larger. For example, given $\tau_{Y}=0.5$, the effective ATFP is 1.102, while the distorted ATFP is 1.041 and the corresponding ATFP loss is about 5.908%.

**Figure 2:**
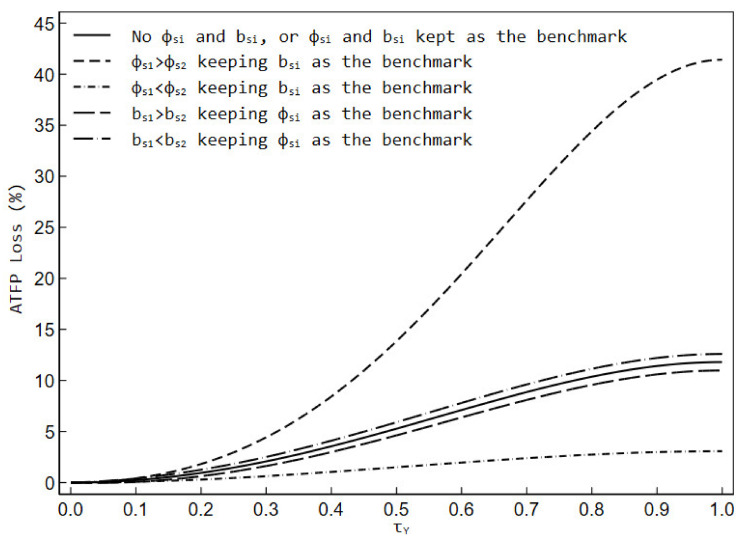
The ATFP loss due to output distortion in different scenarios.

To facilitate readers to compare the simulation results under different scenarios, we have plotted the ATFP losses due to output distortion for various scenarios in Figure 2. It can be seen that factor misallocation does not improve or worsen when firms are subject to the same degree of environmental regulatory shock because the magnitude of ATFP loss is the same as in the absence of environmental regulatory shock. In the benchmark case where the overhead labor share of firms is held, if firm 1 is subject to a stronger environmental regulatory shock than firm 2, the output distortion will result in a larger loss of ATFP, and environmental regulation is not conducive to improving factor misallocation; conversely, if firm 1 is subject to a weaker environmental regulatory shock than firm 2, the ATFP loss due to output distortion will tend to decline, and environmental regulation will help alleviate factor misallocation. Under the baseline scenario where the environmental regulatory shock faced by firms is maintained, if the overhead labor share of firm 1 is larger than that of firm 2, the ATFP loss due to output distortion is lower than the baseline case, and environmental regulation will be able to play a role in mitigating factor misallocation; conversely, if the overhead labor share of firm 1 is smaller than that of firm 2, the ATFP loss due to output distortion is higher than the baseline case, and environmental regulation will likely further exacerbate factor misallocation.

### 4.3. The Impacts of $\tau_{K}$ on ATFP in the Absence/Presence of Environmental Regulatory Shock and Overhead Labor

Table 2 reports the effects of capital distortion on the ATFP in six different scenarios. Similar to the effects of output distortion, the presence of firm-level capital distortion still makes the distorted ATFP lower than the effective ATFP in all scenarios, and the ATFP loss due to capital distortion is different when the environmental regulatory shock is present and absent.

When we did not include environmental regulatory shock and overhead labor inputs (Scenario 1), or set both as the benchmark (Scenario 2), the effective ATFP level remained at 1.118 and the distorted ATFP gradually declined as capital distortion raised. For example, given $\tau_{K}=0.5$, the distorted ATFP was 1.111 and the corresponding ATFP loss was about 0.599%. Again, the results of Scenario 2 mean that the same proportion of environmental regulatory shock or overhead labor share to firms does not change the trend of ATFP.

When the environmental regulatory shock faced by firm 1 was larger than that by firm 2 (Scenario 3), both effective ATFP and distorted ATFP were lower than they would have been without the environmental regulatory shock or with the same degree of shock, and the capital distortion led to a larger decline in ATFP and a larger gap with effective ATFP. For example, given $\tau_{K}=0.5$, the effective ATFP was 1.000, while the distorted ATFP is 0.989 and the corresponding ATFP loss was about 1.115%.

When the environmental regulatory shock faced by firm 1 was smaller than that by firm 2 (Scenario 4), both effective ATFP and distorted ATFP would be higher than they would have been in the absence of the environmental regulatory shock or with the same degree of shock. The rate of decline in ATFP due to capital distortion was relatively slower, and the difference between distorted ATFP and effective ATFP was relatively smaller. For example, given $\tau_{K}=0.5$, the effective ATFP is 1.458, while the distorted ATFP was 1.455 and the corresponding ATFP loss was about 0.192%.

In the case where the environmental regulatory shock to firms was kept as the benchmark, when firm 1 had a higher share of overhead labor than firm 2 (Scenario 5), both effective ATFP and distorted ATFP were higher than they would have been if the firms’ overhead labor were not present or had the same share. At the same time, the rate at which distorted ATFP decreased with a falling degree of capital distortion, and the difference between distorted and effective ATFP tended to be smaller. For example, given $\tau_{K}=0.5$, the effective ATFP is 1.126, while the distorted ATFP is 1.121 and the corresponding ATFP loss is about 0.452%.

Similarly, in the case of the environmental regulatory shock holding benchmark-setting, when firm 1 had a lower share of overhead labor compared to firm 2 (Scenario 6), the effective ATFP and distorted ATFP would both be lower than what they would have been if firms’ overhead labor did not exist or the shares of firm 1 and firm 2 were equal, while distorted ATFP will tend to decline faster with the degree of capital distortion and the gap between it and effective ATFP would become larger. For example, given $\tau_{K}=0.5$, the effective ATFP was 1.102 while the distorted ATFP was 1.094 and the corresponding ATFP loss was about 0.741%.

**Figure 3:**
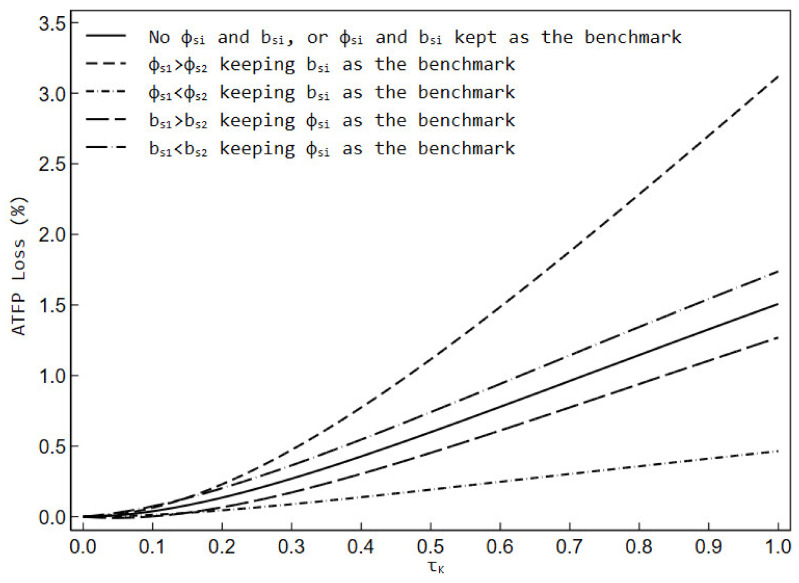
The ATFP loss due to capital distortion in different scenarios.

By the same token, for a better comparative analysis of the results under different scenarios, we plot the ATFP losses due to capital distortion for various scenarios in Figure 3. Again, factor misallocation caused by capital distortion neither improves nor worsens when firms face the same degree of environmental regulatory shock. In the case where firms’ overhead labor share maintains the benchmark, if the environmental regulatory shock to firm 1 is greater than that to firm 2, the capital distortion will lead to a larger ATFP loss, and environmental regulation may further worsen factor misallocation; conversely, the environmental regulatory shock to firm 1 is less than that to firm 2, the ATFP loss due to capital distortion will be narrowed, and environmental regulation will help correct factor misallocation. Under the circumstance that firms face benchmark environmental regulatory shock, if firm 1 has a higher share of overhead labor than firm 2, the ATFP loss due to capital distortion is less than the baseline scenario, and environmental regulation will be able to play a role in mitigating factor misallocation; conversely, if firm 1 has a lower share of overhead labor than firm 2, the ATFP loss due to capital distortion is greater than the baseline scenario, and environmental regulation will likely aggravate factor misallocation.

## 5. Conclusions and Implications

In this paper, we attempted to introduce environmental regulation into a dynamic general equilibrium model to explain its impact on factor misallocation based on the classical theoretical framework proposed by Hsieh and Klenow (2009). From the perspective of total factor productivity, we investigated the changes in the influence of firm-level capital and labor misallocation on industrial aggregate TFP under the condition of environmental regulatory shock. Furthermore, we simplified our general model to a two-firm model and illustrated how environmental regulation affects factor misallocation using the numerical simulation method. The main findings of this paper are as follows.

Firstly, environmental regulation has a significant factor reallocation effect. In the literature review section, we mentioned that there is a small number of empirical studies arguing that environmental regulation can serve the function of allocating factors of production. We reconfirm this view through a theoretical study, which also complements the related research. The numerical simulation showed that environmental regulatory shock did not change the negative impact of factor misallocation on the ATFP, but it had a significant impact on the magnitude of the ATFP loss caused by misallocation. Besides, compared with capital distortion, the ATFP loss due to output distortion is more sensitive to environmental regulatory shock. On the same level of distortion, the environmental regulation shock causes a much larger change in the ATFP loss from output distortion than from capital distortion.

Secondly, the impact of environmental regulation on factor misallocation is uncertain and heterogeneous. Our study shows that the impact of environmental regulatory shock on factor misallocation is not simply positive or negative, and there are both opportunities to mitigate factor misallocation and possibilities to exacerbate factor misallocation, which mainly depends on the firms’ initial factor allocation status and the intensity of the environmental regulatory shock. Compared with the existing literature, this is a new theoretical perspective that enriches the research in the fields of environmental regulation and factor allocation. In terms of quantifying the effect of environmental regulation on factor misallocation, the simulation results show that the ATFP loss due to factor misallocation expands when the intensity of environmental regulatory shock imposed on distorted firms is greater than that of non-distorted firms; conversely, the ATFP loss due to factor misallocation shrinks when the intensity of environmental regulatory shock imposed on distorted firms is less than that of non-distorted firms.

Thirdly, firms’ overhead labor inputs in production can play a role in moderating the factor allocation effects of environmental regulation. According to the theoretical model in Section 3, firms’ overhead labor share is also an important factor affecting distorted ATFP and plays the role of a moderator when environmental regulatory shock acts on the ATFP losses due to output distortion and capital distortion. With the help of numerical simulation, we find that the mitigating effect of environmental regulation on factor mismatch is discounted if, given firms’ exposure to an environmental regulation shock, the ATFP loss due to output distortion or capital distortion is lower than it would have been in the absence of the environmental regulation shock but higher than it would have been if firms’ overhead labor share kept as the benchmark. Conversely, if distorted firms have a lower overhead labor share, the ATFP loss due to output distortion or capital distortion is higher than it would have been in the absence of the environmental regulatory shock but lower than it would have been if firms’ overhead labor share remained as the benchmark, thus helping to dampen the possible worsening of factor market misallocation shocked by environmental regulation.

Based on the above findings, there are two potential policy implications. First, the government can optimally regulate factor markets through differentiated environmental regulation policies. Theoretical simulations show that if a weaker environmental regulation is imposed on firms facing a higher degree of distortion in factor markets, the ATFP loss due to capital or output distortion is much smaller than other environmental regulatory shock scenarios, and environmental regulation can play a positive role in mitigating fac-tor market misallocation, thus providing a feasible option for the government to formulate differentiated environmental regulation policies to optimize factor market allocation. Second, the government can intervene in the factor allocation effect of environmental regulation by guiding firms to determine the appropriate overhead labor input. According to the above findings, if firms face stronger market distortion, reducing their share of overhead labor can provide some disincentive to the possible increase in factor misallocation caused by environmental regulation. Thereby, the government can take appropriate policy measures to guide firms to make reasonable labor input decisions according to the market distortion they face.

Our results also require some caveats. For the sake of simplifying the analysis, our simulations are based on a two-firm model, which does not apply to a multi-firm case, and how to investigate the impact of environmental regulation on factor misallocation in a multi-firm scenario needs to be further explored. Besides, we assumed that firms were subject to the same degree of regulatory shock in simulating how the overhead head played a moderating role in regulating the impact of environmental regulation on factor misallocation, while the moderating effect of different firms facing different regulatory shocks needs to be refined.

## Figures and Tables

**Table 1:** The ATFP and its loss due to output distortion under different scenarios.

	$\tau_{Y}$										
	0	0.1	0.2	0.3	0.4	0.5	0.6	0.7	0.8	0.9	1
Scenario 1. No $\phi_{si}$ and $b_{si}$											
effective ATFP	1.118	1.118	1.118	1.118	1.118	1.118	1.118	1.118	1.118	1.118	1.118
distorted ATFP	1.118	1.115	1.108	1.095	1.080	1.062	1.044	1.027	1.013	1.004	1.000
ATFP Loss (%)	0.000	0.239	0.943	2.072	3.551	5.275	7.102	8.863	10.367	11.413	11.803
Scenario 2. Keep $\phi_{si}$ and $b_{si}$ as the benchmark											
effective ATFP	1.118	1.118	1.118	1.118	1.118	1.118	1.118	1.118	1.118	1.118	1.118
distorted ATFP	1.118	1.115	1.108	1.095	1.080	1.062	1.044	1.027	1.013	1.004	1.000
ATFP Loss (%)	0.000	0.239	0.943	2.072	3.551	5.275	7.102	8.863	10.367	11.413	11.803
Scenario 3. $\phi_{s1}>\phi_{s2}$ keeping $b_{si}$ as the benchmark											
effective ATFP	1.000	1.000	1.000	1.000	1.000	1.000	1.000	1.000	1.000	1.000	1.000
distorted ATFP	1.000	0.996	0.982	0.958	0.922	0.878	0.830	0.784	0.744	0.717	0.707
ATFP Loss (%)	0.000	0.414	1.812	4.427	8.428	13.842	20.440	27.628	34.408	39.466	41.421
Scenario 4. $\phi_{s1}<\phi_{s2}$ keeping $b_{si}$ as the benchmark											
effective ATFP	1.458	1.458	1.458	1.458	1.458	1.458	1.458	1.458	1.458	1.458	1.458
distorted ATFP	1.458	1.457	1.453	1.449	1.443	1.436	1.430	1.424	1.419	1.415	1.414
ATFP Loss (%)	0.000	0.079	0.298	0.629	1.040	1.495	1.957	2.386	2.744	2.988	3.078
Scenario 5. $b_{s1}>b_{s2}$ keeping $\phi_{si}$ as the benchmark											
effective ATFP	1.126	1.126	1.126	1.126	1.126	1.126	1.126	1.126	1.126	1.126	1.126
distorted ATFP	1.126	1.125	1.119	1.108	1.094	1.077	1.059	1.042	1.028	1.018	1.015
ATFP Loss (%)	0.000	0.071	0.623	1.619	2.986	4.620	6.378	8.091	9.565	10.597	10.983
Scenario 6. $b_{s1}<b_{s2}$ keeping $\phi_{si}$ as the benchmark											
effective ATFP	1.102	1.102	1.102	1.102	1.102	1.102	1.102	1.102	1.102	1.102	1.102
distorted ATFP	1.102	1.098	1.089	1.075	1.059	1.041	1.022	1.006	0.992	0.982	0.979
ATFP Loss (%)	0.000	0.402	1.253	2.509	4.097	5.908	7.801	9.608	11.141	12.201	12.595

Data source: The authors provide the data based on theoretical model solving and numerical simulation. The same is as below.

**Table 2:** The ATFP and its loss due to capital distortion under different scenarios.

	$\tau_{K}$										
	0	0.1	0.2	0.3	0.4	0.5	0.6	0.7	0.8	0.9	1
Scenario 1. No $\phi_{si}$ and $b_{si}$											
effective ATFP	1.118	1.118	1.118	1.118	1.118	1.118	1.118	1.118	1.118	1.118	1.118
distorted ATFP	1.118	1.118	1.117	1.115	1.113	1.111	1.109	1.107	1.105	1.103	1.101
ATFP Loss (%)	0.000	0.039	0.135	0.269	0.427	0.599	0.778	0.962	1.145	1.328	1.507
Scenario 2. Keep $\phi_{si}$ and $b_{si}$ as the benchmark											
effective ATFP	1.118	1.118	1.118	1.118	1.118	1.118	1.118	1.118	1.118	1.118	1.118
distorted ATFP	1.118	1.118	1.117	1.115	1.113	1.111	1.109	1.107	1.105	1.103	1.101
ATFP Loss (%)	0.000	0.039	0.135	0.269	0.427	0.599	0.778	0.962	1.145	1.328	1.507
Scenario 3. $\phi_{s1}>\phi_{s2}$ keeping $b_{si}$ as the benchmark											
effective ATFP	1.000	1.000	1.000	1.000	1.000	1.000	1.000	1.000	1.000	1.000	1.000
distorted ATFP	1.000	0.999	0.998	0.995	0.992	0.989	0.985	0.982	0.978	0.974	0.970
ATFP Loss (%)	0.000	0.063	0.230	0.473	0.773	1.115	1.486	1.879	2.285	2.700	3.119
Scenario 4. $\phi_{s1}<\phi_{s2}$ keeping $b_{si}$ as the benchmark											
effective ATFP	1.458	1.458	1.458	1.458	1.458	1.458	1.458	1.458	1.458	1.458	1.458
distorted ATFP	1.458	1.458	1.457	1.456	1.456	1.455	1.454	1.453	1.453	1.452	1.451
ATFP Loss (%)	0.000	0.013	0.045	0.089	0.138	0.192	0.247	0.303	0.358	0.411	0.464
Scenario 5. $b_{s1}>b_{s2}$ keeping $\phi_{si}$ as the benchmark											
effective ATFP	1.126	1.126	1.126	1.126	1.126	1.126	1.126	1.126	1.126	1.126	1.126
distorted ATFP	1.126	1.126	1.126	1.124	1.123	1.121	1.119	1.118	1.116	1.114	1.112
ATFP Loss (%)	0.000	0.002	0.067	0.172	0.304	0.452	0.610	0.774	0.940	1.105	1.269
Scenario 6. $b_{s1}<b_{s2}$ keeping $\phi_{si}$ as the benchmark											
effective ATFP	1.102	1.102	1.102	1.102	1.102	1.102	1.102	1.102	1.102	1.102	1.102
distorted ATFP	1.102	1.101	1.100	1.098	1.096	1.094	1.092	1.090	1.088	1.085	1.083
ATFP Loss (%)	0.000	0.074	0.202	0.364	0.546	0.741	0.941	1.143	1.344	1.543	1.737

## Data Availability

The data used for figures in this study can be obtained by contacting the corresponding author.

## Appendix A. Numerical Simulation Results Using Other Alternative Elasticities of Substitution

Before conducting numerical simulations in Section 4, we set a more conservative value for the output elasticity of substitution between firms, σ=3. The conclusions still hold if we use higher elasticities of substitution—i.e., assuming the differentiated goods are somewhat more homogeneous and easier to substitute. Table A1 and Table A2 report the simulation results when the elasticity of substitution σ=5 (a moderate elasticity), and Table A3 and Table A4 give the simulation results when the elasticity of substitution σ=9 (a more extreme elasticity). It can be seen that the direction of the effects of environmental regulation on factor misallocation in a given scenario is consistent with the findings in the main text, differing only in the change in the absolute magnitude of the effects. The larger the elasticity of substitution, the smaller the magnitude of the ATFP losses caused by output distortion and capital distortion in response to environmental regulatory shocks, so that neither the corrective nor the worsening effects of environmental regulation on factor misallocation tend to become smaller.

**Table A1:** The ATFP and its loss due to output distortion under different scenarios (σ=5).

	$\tau_{Y}$										
	0	0.1	0.2	0.3	0.4	0.5	0.6	0.7	0.8	0.9	1
Scenario 1. No $\phi_{si}$ and $b_{si}$											
effective ATFP	1.015	1.015	1.015	1.015	1.015	1.015	1.015	1.015	1.015	1.015	1.015
distorted ATFP	1.015	1.014	1.011	1.008	1.005	1.003	1.001	1.000	1.000	1.000	1.000
ATFP Loss (%)	0.000	0.116	0.384	0.701	0.997	1.231	1.389	1.478	1.517	1.526	1.527
Scenario 2. Keep $\phi_{si}$ and $b_{si}$ as the benchmark											
effective ATFP	1.015	1.015	1.015	1.015	1.015	1.015	1.015	1.015	1.015	1.015	1.015
distorted ATFP	1.015	1.014	1.011	1.008	1.005	1.003	1.001	1.000	1.000	1.000	1.000
ATFP Loss (%)	0.000	0.116	0.384	0.701	0.997	1.231	1.389	1.478	1.517	1.526	1.527
Scenario 3. $\phi_{s1}>\phi_{s2}$ keeping $b_{si}$ as the benchmark											
effective ATFP	0.841	0.841	0.841	0.841	0.841	0.841	0.841	0.841	0.841	0.841	0.841
distorted ATFP	0.841	0.835	0.818	0.792	0.764	0.740	0.722	0.713	0.708	0.707	0.707
ATFP Loss (%)	0.000	0.677	2.797	6.146	10.059	13.687	16.402	18.014	18.721	18.907	18.921
Scenario 4. $\phi_{s1}<\phi_{s2}$ keeping $b_{si}$ as the benchmark											
effective ATFP	1.416	1.416	1.416	1.416	1.416	1.416	1.416	1.416	1.416	1.416	1.416
distorted ATFP	1.416	1.415	1.415	1.415	1.415	1.414	1.414	1.414	1.414	1.414	1.414
ATFP Loss (%)	0.000	0.008	0.026	0.046	0.065	0.079	0.089	0.095	0.097	0.097	0.098
Scenario 5. $b_{s1}>b_{s2}$ keeping $\phi_{si}$ as the benchmark											
effective ATFP	1.028	1.028	1.028	1.028	1.028	1.028	1.028	1.028	1.028	1.028	1.028
distorted ATFP	1.028	1.028	1.026	1.023	1.020	1.018	1.016	1.015	1.015	1.015	1.015
ATFP Loss (%)	0.000	0.030	0.240	0.521	0.796	1.018	1.171	1.258	1.296	1.306	1.307
Scenario 6. $b_{s1}<b_{s2}$ keeping $\phi_{si}$ as the benchmark											
effective ATFP	0.996	0.996	0.996	0.996	0.996	0.996	0.996	0.996	0.996	0.996	0.996
distorted ATFP	0.996	0.994	0.991	0.987	0.984	0.982	0.980	0.979	0.979	0.979	0.979
ATFP Loss (%)	0.000	0.198	0.521	0.873	1.189	1.433	1.597	1.688	1.726	1.737	1.737

**Table A2:** The ATFP and its loss due to output distortion under different scenarios (σ=5).

	$\tau_{K}$										
	0	0.1	0.2	0.3	0.4	0.5	0.6	0.7	0.8	0.9	1
Scenario 1. No $\phi_{si}$ and $b_{si}$											
effective ATFP	1.015	1.015	1.015	1.015	1.015	1.015	1.015	1.015	1.015	1.015	1.015
distorted ATFP	1.015	1.015	1.015	1.014	1.014	1.013	1.012	1.012	1.011	1.011	1.010
ATFP Loss (%)	0.000	0.018	0.059	0.112	0.170	0.230	0.288	0.345	0.399	0.450	0.498
Scenario 2. Keep $\phi_{si}$ and $b_{si}$ as the benchmark											
effective ATFP	1.015	1.015	1.015	1.015	1.015	1.015	1.015	1.015	1.015	1.015	1.015
distorted ATFP	1.015	1.015	1.015	1.014	1.014	1.013	1.012	1.012	1.011	1.011	1.010
ATFP Loss (%)	0.000	0.018	0.059	0.112	0.170	0.230	0.288	0.345	0.399	0.450	0.498
Scenario 3. $\phi_{s1}>\phi_{s2}$ keeping $b_{si}$ as the benchmark											
effective ATFP	0.841	0.841	0.841	0.841	0.841	0.841	0.841	0.841	0.841	0.841	0.841
distorted ATFP	0.841	0.840	0.838	0.835	0.832	0.828	0.825	0.821	0.817	0.813	0.809
ATFP Loss (%)	0.000	0.088	0.319	0.652	1.055	1.504	1.980	2.471	2.965	3.458	3.942
Scenario 4. $\phi_{s1}<\phi_{s2}$ keeping $b_{si}$ as the benchmark											
effective ATFP	1.416	1.416	1.416	1.416	1.416	1.416	1.416	1.416	1.416	1.416	1.416
distorted ATFP	1.416	1.416	1.416	1.415	1.415	1.415	1.415	1.415	1.415	1.415	1.415
ATFP Loss (%)	0.000	0.001	0.004	0.008	0.011	0.015	0.019	0.023	0.027	0.030	0.033
Scenario 5. $b_{s1}>b_{s2}$ keeping $\phi_{si}$ as the benchmark											
effective ATFP	1.028	1.028	1.028	1.028	1.028	1.028	1.028	1.028	1.028	1.028	1.028
distorted ATFP	1.028	1.028	1.028	1.028	1.027	1.027	1.026	1.026	1.025	1.025	1.024
ATFP Loss (%)	0.000	0.007	0.014	0.050	0.094	0.142	0.190	0.238	0.284	0.328	0.370
Scenario 6. $b_{s1}<b_{s2}$ keeping $\phi_{si}$ as the benchmark											
effective ATFP	0.996	0.996	0.996	0.996	0.996	0.996	0.996	0.996	0.996	0.996	0.996
distorted ATFP	0.996	0.995	0.995	0.994	0.993	0.993	0.992	0.991	0.991	0.990	0.990
ATFP Loss (%)	0.000	0.041	0.101	0.170	0.242	0.313	0.382	0.447	0.509	0.567	0.621

**Table A3:** The ATFP and its loss due to output distortion under different scenarios (σ=9).

	$\tau_{Y}$										
	0	0.1	0.2	0.3	0.4	0.5	0.6	0.7	0.8	0.9	1
Scenario 1. No $\phi_{si}$ and $b_{si}$											
effective ATFP	1.000487	1.000487	1.000487	1.000487	1.000487	1.000487	1.000487	1.000487	1.000487	1.000487	1.000487
distorted ATFP	1.000487	1.000378	1.000213	1.000096	1.000034	1.00001	1.000002	1	1	1	1
ATFP Loss (%)	0	0.010944	0.027448	0.039172	0.045299	0.047791	0.048559	0.048724	0.048744	0.048745	0.048745
Scenario 2. Keep $\phi_{si}$ and $b_{si}$ as the benchmark											
effective ATFP	1.000487	1.000487	1.000487	1.000487	1.000487	1.000487	1.000487	1.000487	1.000487	1.000487	1.000487
distorted ATFP	1.000487	1.000378	1.000213	1.000096	1.000034	1.00001	1.000002	1	1	1	1
ATFP Loss (%)	0	0.010944	0.027448	0.039172	0.045299	0.047791	0.048559	0.048724	0.048744	0.048745	0.048745
Scenario 3. $\phi_{s1}>\phi_{s2}$ keeping $b_{si}$ as the benchmark											
effective ATFP	0.771105	0.771105	0.771105	0.771105	0.771105	0.771105	0.771105	0.771105	0.771105	0.771105	0.771105
distorted ATFP	0.771105	0.762412	0.742278	0.723915	0.713294	0.708831	0.707443	0.707145	0.707108	0.707107	0.707107
ATFP Loss (%)	0	1.140293	3.883673	6.518717	8.104904	8.785585	8.998994	9.044871	9.050515	9.050772	9.050773
Scenario 4. $\phi_{s1}<\phi_{s2}$ keeping $b_{si}$ as the benchmark											
effective ATFP	1.414216	1.414216	1.414216	1.414216	1.414216	1.414216	1.414216	1.414216	1.414216	1.414216	1.414216
distorted ATFP	1.414216	1.414216	1.414215	1.414214	1.414214	1.414214	1.414214	1.414214	1.414214	1.414214	1.414214
ATFP Loss (%)	0	4.29 × 10^−5^	0.000108	0.000153	0.000177	0.000187	0.00019	0.000191	0.000191	0.000191	0.000191
Scenario 5. $b_{s1}>b_{s2}$ keeping $\phi_{si}$ as the benchmark											
effective ATFP	1.015151	1.015151	1.015151	1.015151	1.015151	1.015151	1.015151	1.015151	1.015151	1.015151	1.015151
distorted ATFP	1.015151	1.01513	1.014999	1.014894	1.014837	1.014812	1.014805	1.014803	1.014803	1.014803	1.014803
ATFP Loss (%)	0	0.00213	0.014976	0.025341	0.03103	0.033403	0.034147	0.034308	0.034328	0.034329	0.034329
Scenario 6. $b_{s1}<b_{s2}$ keeping $\phi_{si}$ as the benchmark											
effective ATFP	0.979487	0.979487	0.979487	0.979487	0.979487	0.979487	0.979487	0.979487	0.979487	0.979487	0.979487
distorted ATFP	0.979487	0.979298	0.979102	0.978975	0.978911	0.978886	0.978878	0.978876	0.978876	0.978876	0.978876
ATFP Loss (%)	0	0.019297	0.039267	0.052279	0.058822	0.061426	0.062217	0.062385	0.062406	0.062407	0.062407

**Table A4:** The ATFP and its loss due to output distortion under different scenarios (σ=9).

	$\tau_{K}$										
	0	0.1	0.2	0.3	0.4	0.5	0.6	0.7	0.8	0.9	1
Scenario 1. No $\phi_{si}$ and $b_{si}$											
effective ATFP	1.000487	1.000487	1.000487	1.000487	1.000487	1.000487	1.000487	1.000487	1.000487	1.000487	1.000487
distorted ATFP	1.000487	1.00047	1.000433	1.000391	1.00035	1.000313	1.000279	1.000249	1.000222	1.000199	1.000179
ATFP Loss (%)	0	0.001767	0.005425	0.0096	0.013697	0.017479	0.02087	0.023865	0.026493	0.028792	0.030802
Scenario 2. Keep $\phi_{si}$ and $b_{si}$ as the benchmark											
effective ATFP	1.000487	1.000487	1.000487	1.000487	1.000487	1.000487	1.000487	1.000487	1.000487	1.000487	1.000487
distorted ATFP	1.000487	1.00047	1.000433	1.000391	1.00035	1.000313	1.000279	1.000249	1.000222	1.000199	1.000179
ATFP Loss (%)	0	0.001767	0.005425	0.0096	0.013697	0.017479	0.02087	0.023865	0.026493	0.028792	0.030802
Scenario 3. $\phi_{s1}>\phi_{s2}$ keeping $b_{si}$ as the benchmark											
effective ATFP	0.771105	0.771105	0.771105	0.771105	0.771105	0.771105	0.771105	0.771105	0.771105	0.771105	0.771105
distorted ATFP	0.771105	0.770048	0.767357	0.763685	0.759542	0.75528	0.751123	0.747199	0.743571	0.740261	0.737266
ATFP Loss (%)	0	0.137301	0.488497	0.971617	1.522417	2.095265	2.660278	3.199436	3.703012	4.166756	4.589863
Scenario 4. $\phi_{s1}<\phi_{s2}$ keeping $b_{si}$ as the benchmark											
effective ATFP	1.414216	1.414216	1.414216	1.414216	1.414216	1.414216	1.414216	1.414216	1.414216	1.414216	1.414216
distorted ATFP	1.414216	1.414216	1.414216	1.414216	1.414215	1.414215	1.414215	1.414215	1.414215	1.414215	1.414215
ATFP Loss (%)	0	6.95 × 10^−6^	2.13 × 10^−5^	3.77 × 10^−5^	5.37 × 10^−5^	6.85 × 10^−5^	8.18 × 10^−5^	9.35 × 10^−5^	1.04 × 10^−4^	1.13 × 10^−4^	1.21 × 10^−4^
Scenario 5. $b_{s1}>b_{s2}$ keeping $\phi_{si}$ as the benchmark											
effective ATFP	1.015151	1.015151	1.015151	1.015151	1.015151	1.015151	1.015151	1.015151	1.015151	1.015151	1.015151
distorted ATFP	1.015151	1.015166	1.015153	1.015127	1.015099	1.015071	1.015044	1.01502	1.014998	1.014979	1.014962
ATFP Loss (%)	0	0.000109	0.001457	0.002362	0.005175	0.007969	0.010585	0.012965	0.015096	0.01699	0.018666
Scenario 6. $b_{s1}<b_{s2}$ keeping $\phi_{si}$ as the benchmark											
effective ATFP	0.979487	0.979487	0.979487	0.979487	0.979487	0.979487	0.979487	0.979487	0.979487	0.979487	0.979487
distorted ATFP	0.979487	0.97944	0.979382	0.979326	0.979274	0.979228	0.979187	0.979152	0.979122	0.979096	0.979073
ATFP Loss (%)	0	0.004822	0.010671	0.016459	0.021774	0.026493	0.030617	0.034196	0.037294	0.039976	0.042303
